# Supplementary material for: Polyunsaturated fatty acids and their endocannabinoid-related metabolites activity at human TRPV1 and TRPA1 ion channels expressed in HEK-293 cells
Source: PeerJ. 2025 Mar 24;13:e19125. doi: 10.7717/peerj.19125 (PMC11949107; doi:10.7717/peerj.19125)
Supplement: Supplemental Information 2 [file peerj-13-19125-s002.docx]

**Supplementary Table 2:** PUFAs and their endocannabinoid metabolites included in this study, indicating previously studied metabolites and novel findings in HEK-293 TRPV1 and TRPA1 cells.

| **PUFAs and their metabolites** | **TRPV1** | | **TRPA1** | |
| --- | --- | --- | --- | --- |
|  | **Previous studies** | **Novel Findings** | **Previous studies** | **Novel Findings** |
| **PUFAs** |  |  |  |  |
| EPA | Yes | No | Yes | No |
| DHA | Yes | No | Yes | No |
| γ-LA | No | Yes | No | Yes |
| 9:3:1 ratio | No | Yes | No | Yes |
| **PUFA metabolites** | | | | |
| EPEA | No | Yes | No | Yes |
| DHEA | No | Yes | No | Yes |
| γ-LEA | No | Yes | No | Yes |
| AEA | Yes | No | Yes | No |
| 2-AG | Yes | No | Yes | No |
| 2-AG ether | Yes | No | Yes | No |
| 2-LG | Yes | No | Yes | No |
| NADA | Yes | No | Yes | No |
