# Supplementary material for: Polyunsaturated fatty acids and their endocannabinoid-related metabolites activity at human TRPV1 and TRPA1 ion channels expressed in HEK-293 cells
Source: PeerJ. 2025 Mar 24;13:e19125. doi: 10.7717/peerj.19125 (PMC11949107; doi:10.7717/peerj.19125)
Supplement: Supplemental Information 3 [file peerj-13-19125-s003.docx]

**Supplementary Figure 1.**Traces of PUFAs and their endocannabinoid related metabolites at 10 µM in HEK-293 empty vector cell.
