# Supplementary material for: Polyunsaturated fatty acids and their endocannabinoid-related metabolites activity at human TRPV1 and TRPA1 ion channels expressed in HEK-293 cells
Source: PeerJ. 2025 Mar 24;13:e19125. doi: 10.7717/peerj.19125 (PMC11949107; doi:10.7717/peerj.19125)
Supplement: Supplemental Information 4 [file peerj-13-19125-s004.docx]

**Supplementary Figure 2.** Traces of Ionomycin and Capsaicin in HEK-293 TRPV1 expressing cells.
